# Habitat Complexity in Aquatic Microcosms Affects Processes Driven by Detritivores

**DOI:** 10.1371/journal.pone.0165065

**Published:** 2016-11-01

**Authors:** Lorea Flores, R. A. Bailey, Arturo Elosegi, Aitor Larrañaga, Julia Reiss

**Affiliations:** 1 INRA, UMR 1224, Ecologie Comportementale et Biologie des Populations de Poissons, Aquapôle, quartier Ibarron, 64310 Saint-Pée sur Nivelle, France; 2 School of Mathematical Sciences, Queen Mary University of London, London E1 4 NS, United Kingdom; 3 School of Mathematics and Statistics, University of St Andrews, St Andrews, Fife, KY16 9SS, United Kingdom; 4 Laboratory of Stream Ecology, Dept. of Plant Biology and Ecology, Fac. of Science and Technology, University of the Basque Country, UPV/EHU PO Box 644; 48080 Bilbao, Spain; 5 Department of Life Sciences, Whitelands College, University of Roehampton, London SW15 4JD, United Kingdom; Towson University, UNITED STATES

## Abstract

Habitat complexity can influence predation rates (e.g. by providing refuge) but other ecosystem processes and species interactions might also be modulated by the properties of habitat structure. Here, we focussed on how complexity of artificial habitat (plastic plants), in microcosms, influenced short-term processes driven by three aquatic detritivores. The effects of habitat complexity on leaf decomposition, production of fine organic matter and pH levels were explored by measuring complexity in three ways: 1. as the presence vs. absence of habitat structure; 2. as the amount of structure (3 or 4.5 g of plastic plants); and 3. as the spatial configuration of structures (measured as fractal dimension). The experiment also addressed potential interactions among the consumers by running all possible species combinations. In the experimental microcosms, habitat complexity influenced how species performed, especially when comparing structure present *vs*. structure absent. Treatments with structure showed higher fine particulate matter production and lower pH compared to treatments without structures and this was probably due to higher digestion and respiration when structures were present. When we explored the effects of the different complexity levels, we found that the amount of structure added explained more than the fractal dimension of the structures. We give a detailed overview of the experimental design, statistical models and R codes, because our statistical analysis can be applied to other study systems (and disciplines such as restoration ecology). We further make suggestions of how to optimise statistical power when artificially assembling, and analysing, ‘habitat complexity’ by not confounding complexity with the amount of structure added. In summary, this study highlights the importance of habitat complexity for energy flow and the maintenance of ecosystem processes in aquatic ecosystems.

## Introduction

The physical structure of the environment (e.g. habitat complexity) shapes how species co-exist and interact and thus drive ecosystem processes [1]. For example, the fractal dimension of aquatic plants can influence how many individuals [2] and species [3] can colonise a given surface. ‘Habitat complexity’ is a multifaceted term that is widely used in different ecological disciplines such as restoration ecology or conservation. Many (contrary) definitions of the term exist (e.g. [4–6]), but in its ‘simplest’ form, the term describes the attributes of a physical structure that is used as a habitat by organisms [4]. In aquatic habitats, physical structure can be ‘complex’ in terms of its diversity or size for example [4], and in the following we consider ‘presence *vs*. absence’, ‘amount’ and ‘spatial arrangement’ of structure exclusively. These three measures are all aspects of what makes structure ‘complex’, ‘diverse’ or ‘heterogeneous’. The amount and arrangement of habitat structure can affect ecosystem functioning because, over longer time scales, complexity increases the possibilities for niche differentiation (i.e. ‘maximises’ species richness and abundance). An excellent example for the latter is a study by Cardinale and colleagues who manipulated substrate heterogeneity, by either narrowing or widening the gravel size distribution in a small stream, which greatly affected biofilm metabolism [7].

Importantly, habitat complexity has a direct effect on species interactions even over short time scales. For example, structures provide refuge against predation and their amount and/or fractal dimension can generally change both the predation rate and the functional response patterns of predators, as experimentally shown for the copepod *Eucyclops serrulatus* [8], the centipede *Lithobius mutabilis* [9] or the bluegill sunfish [10] for instance. Changes in predator-prey dynamics are an important mechanism that underlies the ‘short-term’ effects of habitat complexity on communities. Similarly, other species interactions could also change with habitat complexity. For example, it is conceivable that different consumer species feeding on the same food resource interact less when habitat complexity is high or that complexity influences the overall performance of an assemblage because species can operate in their ‘optimal’ dimensional environment. However, to our knowledge, there are no studies testing this hypothesis, but there is strong evidence that species are adapted (foraging and feeding) to the dimensionality of their environment [11].

Primary consumers drive a range of ecosystem processes and their effects on food resource depletion typically receive most attention. For example, controlled laboratory experiments with aquatic decomposers have shown that species identity (e.g. [12]), as well as body mass and biomass [13,14] of species, drive leaf decomposition rates. These tightly controlled experiments with small species pools can inform general ecological theory and have been used in biodiversity-ecosystem functioning (B-EF) research. For instance, when different leaf consumer species are in combination with each other they do not show facilitation—their effects on a single process (e.g. leaf decomposition rate) are often additive [14,15] but abiotic factors, such as temperature, can change the outcomes of B-EF experiments [15,16]. Similarly, habitat complexity could alter additive effects of consumers if species have different ways of ‘using’ habitat structure, especially if they differ in terms of body size. For example, species with very different body sizes can perceive the same structure as two- or three dimensional. In addition, more complex physical structure could have a positive effect on overall performance of an assemblage because species can feed in their optimum environment (in analogy to their optimum temperature). The amount or arrangement of physical structure could, therefore, influence consumer effects and could be an important predictor to consider in B-EF research, similarly to body size and temperature [16,17].

Using aquatic microcosms with and without plastic plants, we tested whether different levels of habitat complexity modulated the effects of three detritivore species on three response variables–both when feeding in isolation and when feeding together (all possible monocultures and combinations). In previous experiments, we found that two of these species, *Asellus* and *Gammarus*, did not interact when they were in combination with each other [14], but here we added a third crustacean, *Cyclops*, which is much smaller than the other two species and should feed in a different fashion and also perceive structure differently. We measured habitat complexity in three ways: 1. ‘structure present *vs*. structure absent’, 2. ‘amount (i.e. mass) of structures’ and 3. ‘fractal dimension of structures’.

We tested two hypotheses: firstly, we expected that processes associated with leaf decomposition would increase with increasing complexity because more complex environments generally enhance foraging and feeding [11]. Secondly, we hypothesised that complexity would be more important for processes than species interactions or identity *per se*. This means that we expected processes to be additive (mono- and polycultures do not perform in an unexpected manner) but that processes are altered by complexity because species are more active when they feed in a more complex environment. Our overall aim was to show that habitat complexity can influence processes driven by detritivores and we found evidence for this—two out of the three processes we measured changed when complexity was altered.

## Methods

### Set-up and organisms

Individual microcosms (beakers, 9.5 cm width, 11.5 cm high) served as experimental units. Air-dried alder leaves (*Alnus glutinosa* L.) were weighed, added to each microcosm (2g), and conditioned in 500 mL water (1 part of filtered pond water [GF/C filter] to 5 parts of tap water) for 7 days when the water was renewed and animals were introduced to the microcosms. Every microcosm was served by one air-stone and covered with cling film to reduce evaporation. The experiment was run in a temperature controlled room (15°C) with a 14 h light: 10 h dark cycle for 29 days.

Three invertebrate detritivore species, the amphipod *Gammarus pulex* (L.), the isopod *Asellus aquaticus* (L.) and the copepod *Cyclops viridis* (Jurine) were collected in April 2013 from a small pond on the university grounds (the Swell, Whitelands College, London). These three species often co-occur (e.g. [18]) and while all species compete for the leaf resources added [13–15], we expected the copepod *Cyclops* (much smaller than *Asellus* and *Gammarus*) to profit from the presence of other species. Copepods can feed on faeces [19] and they mainly feed on the microbes decomposing leaves rather than the leaves themselves (as *Asellus* and *Gammarus* do).

### Biodiversity and Habitat Complexity Levels

To create different biodiversity levels, we used monocultures and all possible polycultures of our three species. The abbreviations for the cultures are as follows: A, G, C, AG, AC, GC and AGC (where A is *Asellus*, G is *Gammarus* and C is *Cyclops*).

**Fig 1:**
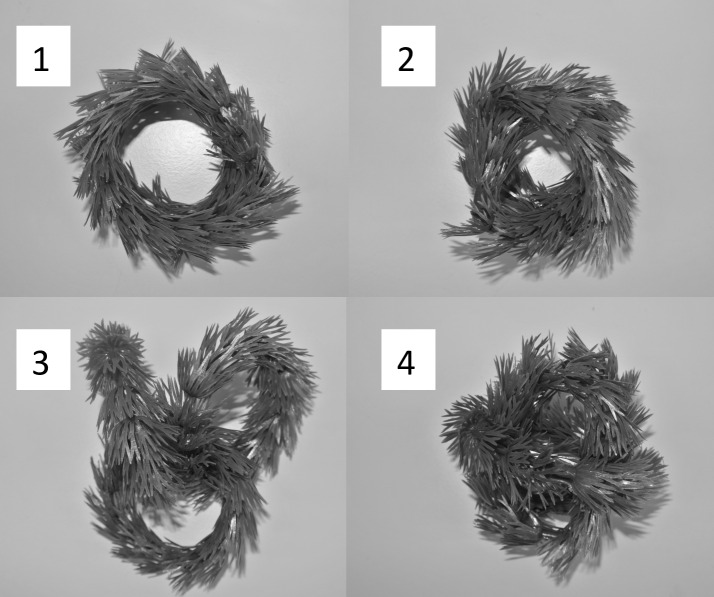
Photographs of the structures used to create habitat complexity in microcosms with ‘structure present’. The basic unit of each structure was a plastic plant strip (mimicking *Ceratophyllum* spp.), joined up as a ring (~ 8cm in diameter) and four levels of fractal dimension were created with them: 1) level 1 consisted of two rings aligned, with a fractal dimension (D) of 1.77; 2) level 2 consisted of two rings twisted into each other (D = 1.80); 3) level 3 consisted of three rings locked together (D = 1.81) and 4) level four was a ball made from 3 rings together (D = 1.83). This design therefore also gave two levels of ‘amount of structure’ - 3 g for complexity level 1 and 2 and 4.5 g for complexity level 3 and 4.

All microcosms had 2 g of leaves as a food resource, which represented structure invertebrates were able to hide in. For our manipulation of complexity, however, we ignored this ‘baseline complexity’ and ‘complexity’ as defined here was manipulated using plastic plant strips mimicking *Ceratophyllum* spp. (Code No. FRF 491, Fish are Fun®). These strips can be joined together so they form a ring (Fig 1). When manipulating habitat complexity levels, we aimed to disentangle the effects that would be due to 1) the presence *vs*. absence of structure (i.e. the plastic plant rings), 2) the amount of structure (i.e. number and mass of plastic rings) and 3) those due to the spatial configuration (fractal dimension) of structures in the microcosm. Therefore, we ran microcosms without any plastic plants added, (called ‘structure absent’) and then we ran other microcosms that received plastic plants and this produced another four levels of habitat complexity (these ‘structure’ microcosms were called ‘complexity level 1, 2, 3 and 4’). These four levels were created with the rings, varying ring number and arrangement (Fig 1). For levels 1 and 2 we used two rings, weighing 3 g together, and for levels 3 and 4 we used three, weighing 4.5 g together (Fig 1). We further calculated the fractal dimension of each of the four structures following [8], as the fractal dimension D, calculated from a two-dimensional picture of the introduced pattern using the grid method [20]. The software used was IMAGEJ [21] and FracLac v.1.2 [22]. The fractal dimension D of the patterns ranged from 1.77 for the lowest complexity to 1.83 for the highest complexity (Fig 1). When structure was absent, we gave this a fractal dimension of zero.

We measured temperature at the beginning of the experiment and after 24 h (period of leaf leaching) to check that the structures were not warming the microcosms due to their opacity (*i*.*e*. confounding complexity with temperature), and indeed this was not the case.

### Treatments

**Fig 2:**
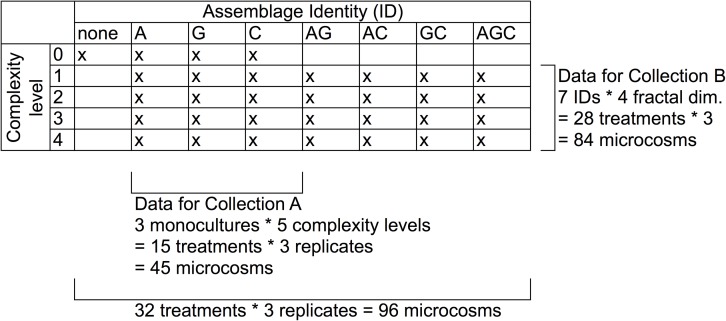
Experimental design of the experiment addressing the effect of complexity level and species combinations on three response variables. In total, there were five complexity levels including structure absent and levels 1–4 (created with plastic plants); and 7 assemblage identities (A is *Asellus*, G is *Gammarus* and C is *Cyclops*). Our design was not fully factorial: only monocultures were run with structure absent. We therefore divided the dataset in two sub-sets and used two different collections of statistical models on them (collection A and B).

In total we ran 32 treatments (Fig 2) but these did not represent a fully factorial set-up with all combinations of complexity levels and biodiversity levels due to space constraints in the environment chamber. Di- and tri- cultures were not run with ‘structure absent’ (Fig 2) but all other possible combinations of complexity and biodiversity level were run, in addition to a control (Fig 2).

All treatments were replicated three times (run in three blocks), which resulted in 96 microcosms in total (Fig 2, S1 Table). The three ‘blocks’ were three shelves in the temperature controlled room and we made sure that whilst microcosm placement was random on a shelf, each shelf contained exactly all 32 treatments.

### Adjusting for metabolic capacity in the microcosms

All animals were photographed at 100X before they were put into the microcosms. The body length of each individual was then measured with the image analysis software Image-Pro® Plus (Media Cybernetics, Inc.). The body mass (M) of all crustaceans (in mg dry mass for *Asellus* and *Gammarus* and μg dry mass for *Cyclops*) was estimated from their body length (L) using published equations (*Asellus* and *Gammarus* equations after Reiss and colleagues [13] and *Cyclops* after Dumont and colleagues [23]:

$$\begin{array}{l} Asellus\;aquaticus\;\left(mg,\;mm\right):\;log_{10}\left(M\right)=2.652\;*\;log_{10}\left(L\right)-1.841\\ Gammarus\;pulex\;\left(mg,\;mm\right):\;log_{10}\left(M\right)=3.015\;*\;log_{10}\left(L\right)-2.242\\ Cyclops\;viridis\;\left(\mu g,\;\mu m\right):\;M=4.9\;*\;10^{-8}\;*\;L^{2.75} \end{array}$$

(equations given as in the publication).

Instead of adjusting for abundance or biomass in the microcosms, we decided to adjust for the metabolic capacity of each species in the treatments, because metabolic capacity drives feeding activity and, for *Gammarus* and *Asellus*, we found previously that decomposition followed a ¾ scaling law with body mass [14] which is consistent with the assumption that metabolic power explains decomposition. We calculated metabolic capacity (*MC*; in mg) of each monoculture after Perkins and colleagues [15], based upon general allometric body mass—metabolism relationships:

$$MC=\sum \left(M^{0.75}\right)$$

where *M* is individual dry mass (mg) of each individual. The ¾ exponent (i.e. 0.75) describes a general relationship between basal metabolic rate and body mass, and has been applied to describe the allometric scaling of basal metabolic rate across a wide range of organisms (see [24,25]). Because small animals ingest more food in relation to their own body mass compared to larger organisms, adjusting for the metabolic potential resulted in a smaller proportion of *Cyclops vs*. *Asellus* individuals than adjusting for the biomass. Thus, taking into account that the total metabolic capacity of each combination would be the sum of the metabolic capacity of all the animals in the assemblage, we adjusted all monocultures to the same metabolic potential by adding either 218 *Cyclops* (1.6 mm in length on average), 15 *Gammarus* (6.4 mm in length on average), or 12 *Asellus* (6.6 mm in length on average) to the respective monoculture microcosm. We used roughly ½ and ⅓ of these numbers in the di- and tri-cultures respectively (*i*.*e*. 109, 8 and 6 in di-cultures and 73, 5 and 4 in tri-cultures), amounting to the same metabolic capacity for all microcosms.

### Response variables

We measured three response variables: leaf breakdown, production of fine particulate organic matter (FPOM [mainly faeces]) and pH. Every four days, pH was measured in the microcosms to account for microbial and invertebrate activity (respiration). High respiration means high levels of CO_2_, which lowers the pH through the carbonate buffer system (as CO_2_ dissolves in H_2_O, more protons are released), therefore we expected pH would decrease with microbial and invertebrate activity. After the experiment had been terminated, leaf material was dried at 80°C for three days and weighed. Leaf mass loss was then calculated after correcting for losses caused by leaching and expressed as leaf mass loss in g per day (see [14]). We used the pH measured on day 7 in the microcosms as our response variable (but see S2 Table to show that the other pH measurements delivered the same result). In addition, FPOM in the microcosms, consisting of faeces and finely shredded leaf material (<1mm diameter), was dried and weighed separately (we used the amount produced [in g] per day as our response variable).

### Statistical Analyses

To assess the effect of habitat complexity and species richness on all three response variables, we performed a classical analysis of variance (ANOVA). This involves fitting several different linear models at the same time. Because much of the (biological) literature calls one ANOVA a ‘model’ we want to point out that we use ‘model’ in a different sense. For example, `Amount of Structure' and `Fractal Dimension' are two of our models. The former is a special case of the latter, so the factor ‘Fractal Dimension’ is said to be nested in ‘Amount of Structure.’ The words ‘factor’ or ‘predictor’ are often used in the literature as synonyms for what we call models, but that terminology is not appropriate here because some of our models have more than one factor or predictor (we fit nested factors and interactions). The single ANOVA for a collection of models enables us to test hypotheses and hence to choose the smallest model that fits the data adequately (see also [14, 26–28]. We had seven ‘basic’ models: ‘Block’, ‘Richness’, ‘Monoculture Identity’, ‘Assemblage Identity’ and, because the complexity of the plastic plants can be described by more than one metric and our aim was to explore the different ways habitat structure can be measured, ‘Structure’, ‘Amount’ and ‘Fractal Dimension’. When these basic models are nested or we test for interactions, we name the models accordingly (e.g. ‘Monoculture Identity nested in Richness’, Table 1).

**Table 1:** Statistical models fitted in two collections of models.

Model name		Model tests for the effects of.	Para-meters	df	R output
***Collection A*: *Models for monocultures with structure present or absent***					
Block	Block	block	3	2	Block
Structure	S	structure present *vs*. structure absent	2	1	S
Monoculture Identity	M	A, G and C	3	2	M
Amount nested in Structure	A(S)	3g, 4.5g in structure, no structure	3	1	S:A
Interaction S and M	S*M	interaction between the predictors	6	2	S:M
Fractal dimension nested in A(S)	F(A(S))	fractal dimension (1,2,3,4) in amount (3g, 4.5g)	5	2	S:A:F
Interaction S, A and M	A(S)*M	interaction between the predictors	9	2	S:A:M
Interaction S, A, F and M	F(A(S))*M	interaction between the predictors	15	4	S:A:F:M
***Collection B*: *Models for a range of complexity levels and number of species***					
Block	Block	block	3	2	Block
Amount of structure	A	is the amount of structures more important than its fractal dimension?	2	1	A
Richness	R	richness of species (1, 2 and 3)	3	2	R
Type	T	covariates	3	2	x1 and x2
Assemblage Identity	ID(R, T)	monocultures and all possible polycultures	7	2	ID
Fractal dimension nested in A	F(A)	fractal dimension (1, 2, 3 and 4)	4	2	A:F
Interaction A and R	A*R	interaction between the predictors	6	2	A:R
Interaction A and T	A*Type	interaction between the predictors	6	2	A:x1and A:x2
Interaction A and ID	A*ID(R, T)	interaction between the predictors	14	2	A:ID
Interaction F and R	F(A)*R	interaction between the predictors	12	4	A:F:R
Interaction F and T	F(A)*T	interaction between the predictors	12	4	A:F:x1 and A:F:x2
Interaction F and ID	F(A)*ID(R, T)	interaction between the predictors	28	4	A:F:ID

Statistical model collection A was used on data from 45 microcosms (15 treatments, 3 replicates) containing monocultures and structure absent or present. All models in collection A are tested in R simultaneously using the command 'Block + (S/A/F)*M'. Collection B was used on data from 84 microcosms (28 treatments, 3 replicates) containing mono- and polycultures and different levels of habitat complexity. All models in collection B were tested in R using the command ‘aov(response ~Block + (A/F)*(R + x1 + x2 +ID))'.

We did not have a fully factorial design, because di- and tri- cultures were not run at ‘structure absent’ (Fig 2). Therefore, we performed two separate ANOVAs, each testing a different collection of models (Collections A and B, Table 1) on a different data-subset (Fig 2).

Collection A (Table 1) can answer the overall question ‘what is the effect of structure compared to ‘no structure’?’, comparing two extreme cases of ‘habitat complexity’. We tested a collection of linear models on data from 15 treatments (3 species [A, G, C] * 5 complexity levels), replicated 3 times, giving 45 microcosms (Fig 2). Collection A simultaneously tested for ‘Monoculture Identity’, ‘Block’, ‘Structure’, ‘Amount’ (nested in ‘Structure’) and ‘Fractal Dimension’ (nested in ‘Amount’) (Table 1).

Collection B (Table 1) was used on data from 84 microcosms (28 treatments, 3 replicates) containing mono- and polycultures and different levels of habitat complexity, but excluding those with structure absent. Collection B can provide answers to the question ‘does an increase in complexity level (measured as amount and fractal dimension) change species interactions and their effect on the food resource?’. We tested this collection of models on 28 treatments (7 assemblage identities [A, G, C, AG, AC, GC, AGC] * 4 fractal dimensions [1, 2, 3, 4] (those included two ‘amount’ levels) = 84 microcosms; Fig 2). To test for complexity and species richness effects, collection B tested for ‘Block’, ‘Amount of Structure’, ‘Fractal Dimension’ (nested in Amount), ‘Richness’, ‘Assemblage Identity’ (nested in Richness), and for all interactions of these predictors.

To test if Assemblage Identity effects were due to additive performance of individual species or a case of facilitation (or other inter-species interaction), we included one more model in Collection B—a model we previously dubbed ‘Type’ [14,26]. The ‘Type’ model assumes that each species (or type) has a unique effect, which provokes a characteristic response irrespective of whether the type is combined with other types or not. The response simply depends on additive effects of types (i.e. species): for monoculture A it should be *α*_1_; for di-culture AG it should be (*α*_1_ + *α*_2_)/2; and for tri-culture AGC it should be (*α*_1_ + *α*_2_ + *α*_3_)/3 (see S1 File for an explanation of how this translates to fitted values for this model). Importantly (and unlike what was done in [14]), the metabolic power of species *i* was multiplied by the number of organisms of species i present in a given assemblage identity for *i* = 1, 2, 3 to define covariates *x*_1_, *x*_2_ and *x*_3_. Note that the model `Assemblage Identity' is now nested in `Type' as well as in `Richness'. Allowing the parameters *α*_*i*_ to change according to the levels of other predictors gives interaction models such as Amount*Type.

All statistical tests were carried out using the statistical software R 2.5.1 [29]. The R output is straightforward for Collections A and B. Only collection B needs a little additional computation, because the R output gives the sums of squares for *x*_1_ and *x*_2_ separately: for the final ANOVA table, the SS values *x*_1_ and *x*_2_ must be added to obtain SS for the model `Type'. This must also be done for models that include an interaction between Type and another predictor (see S1 File for R code and worked example).

## Results

### Complexity measured as ‘structure present *vs*. structure absent’ in monocultures

**Fig 3:**
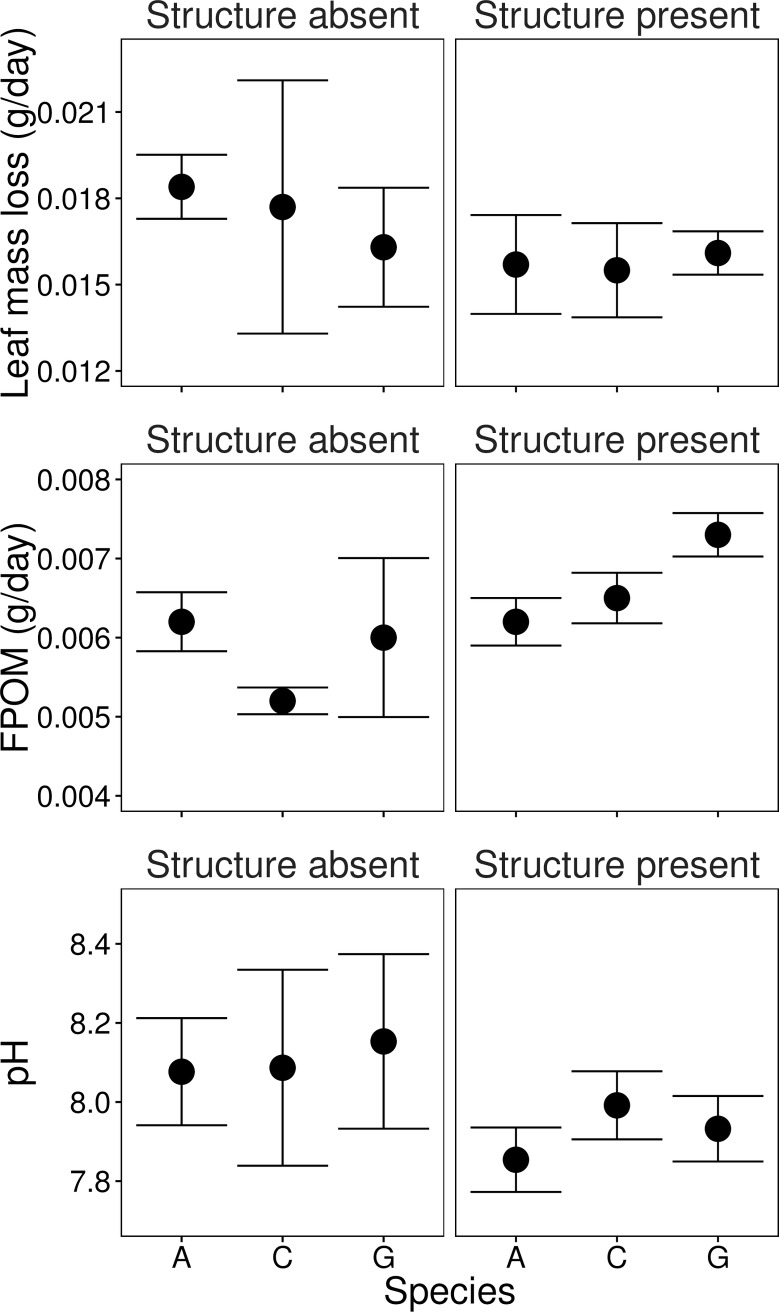
Processes (leaf decomposition; FPOM production and pH) driven by monocultures. Monoculture microcosms of *Asellus* (A), *Gammarus* (G) and *Cyclops* (C) were run with structure absent (left hand panels) and structure (plastic plants) present (right hand panels). Averages for both FPOM and pH values are significantly different for structure absent compared to microcosms with structure present. The average values for leaf decomposition (g/day), FPOM (g/day) and pH (on day 7) were (±SE): 0.0174 (±0.0015), 0.0058 (±0.0003) and 8.11 (±0.11), respectively, when structure was absent and 0.0157 (±0.0008); 0.0067 (±0.0001) and 7.93 (±0.05), respectively, when structure was present.

When we compared the effects of structure present *vs*. structure absent in the single-species cultures (i.e. the monocultures, Fig 3), we found that leaf mass loss was not affected by the presence of structure, nor did a particular species perform better than another one (i.e. models ‘Monoculture’ and ‘Structure’ were not significant, Table 2, Fig 3). However, significantly more FPOM was produced in monocultures where structures (comprising complexity levels 1 to 4) were present compared to those where plastic plants were absent (Table 2, Fig 3). The presence of structure also had a significant effect on pH–pH was significantly lower in the microcosms with structure than in the other monocultures that had not received any plastic plants (Table 2, Fig 3). For both these responses, the amount of structure added to the microcosm or the fractal dimension of the structures did not explain more than the model ‘Structure’ (Table 2).

**Table 2:** Analysis of variance for three response variables.

		Leaf mass loss				FPOM production				pH			
Source	df	SS	MS	F	P	SS	MS	F	P	SS	MS	F	P
***Collection A*: *Structure absent vs*. *structure present***													
Block	2	0.0002064	0.0001032	4.05	**0.03**	0.0000005	0.0000002	0.19	0.83	2.9960	1.4980	98.71	**<0.001**
S	1	0.0000208	0.0000208	0.81	0.37	0.0000054	0.0000054	4.14	**0.05**	0.2318	0.2318	15.28	**<0.001**
M	2	0.0000008	0.0000004	0.02	0.98	0.0000071	0.0000036	2.73	0.08	0.0989	0.0495	3.26	**0.05**
A(S)	1	0.0000005	0.0000005	0.02	0.89	0.0000001	0.0000001	0.10	0.76	0.0003	0.0003	0.02	0.89
S*M	2	0.0000078	0.0000039	0.15	0.86	0.0000028	0.0000014	1.07	0.36	0.0257	0.0128	0.85	0.44
F(A(S))	2	0.0000090	0.0000045	0.18	0.84	0.0000007	0.0000004	0.28	0.76	0.0346	0.0173	1.14	0.33
A(S)*M	2	0.0000369	0.0000184	0.72	0.49	0.0000028	0.0000014	1.08	0.35	0.0050	0.0025	0.17	0.85
F(A(S))*M	4	0.0000018	0.0000005	0.02	1.00	0.0000017	0.0000004	0.33	0.85	0.0683	0.0171	1.13	0.36
Error	28	0.0007137	0.0000255			0.0000365	0.0000013			0.4249	0.0152		
Total	44												
***Collection B*: *Range of complexity levels***													
Block	2	0.000284	0.000142	4.01	**0.02**	0.00000175	0.00000087	0.898	0.41	4.820	2.4101	114.89	**<0.001**
A	1	0.000054	0.000054	1.52	0.22	0.00000004	0.00000004	0.038	0.85	0.090	0.0900	4.29	**0.04**
R	2	0.000008	0.000004	0.11	0.90	0.00000251	0.00000125	1.29	0.28	0.058	0.0289	1.38	0.26
T	2	0.000023	0.000012	0.33	0.72	0.00001051	0.00000526	5.41	**0.007**	0.101	0.0505	2.40	0.10
ID(R, T)	2	0.000044	0.000022	0.61	0.54	0.00000071	0.00000036	0.365	0.70	0.030	0.0148	0.71	0.50
F(A)	2	0.000149	0.000075	2.11	0.13	0.00000137	0.00000069	0.705	0.50	0.002	0.0008	0.04	0.96
A*R	2	0.000056	0.000028	0.79	0.46	0.00000013	0.00000007	0.068	0.93	0.060	0.0298	1.42	0.25
A*T	2	0.000005	0.000003	0.08	0.93	0.00000174	0.00000087	0.90	0.41	0.002	0.0010	0.05	0.95
A*ID(R, T)	2	0.000090	0.000045	1.27	0.29	0.00000127	0.00000064	0.655	0.52	0.009	0.0046	0.22	0.80
F(A)*R	4	0.000110	0.000027	0.77	0.55	0.00000478	0.00000119	1.228	0.31	0.125	0.0313	1.49	0.22
F(A)*T	4	0.000022	0.000005	0.15	0.96	0.00000183	0.00000046	0.47	0.76	0.033	0.0083	0.39	0.81
F(A)*ID(R, T)	4	0.000081	0.000020	0.57	0.69	0.00000067	0.00000017	0.172	0.95	0.056	0.0139	0.66	0.62
Error	54	0.001913	0.000035			0.00005251	0.00000097			1.133	0.0210		
Total	83												

Statistical model collection A was used on data from 45 microcosms (15 treatments, 3 replicates) containing monocultures and structure present or absent. Collection B was used on data from 84 microcosms (28 treatments, 3 replicates) containing mono- and polycultures and different levels of habitat complexity. The model names are given as their abbreviation: M = Monoculture Identity; A = Amount of structure; R = Richness, T = Type, ID = Assemblage Identity; F = Fractal Dimension; brackets indicate nesting and stars an interaction term.

The identity of the species (model ‘Monoculture’, Table 2, Collection A) was only important for the response pH, but not for leaf decomposition or FPOM. There was however, an indication that the three species had different FPOM production (model ‘Monoculture’ almost significant, Table 2) and indeed *Gammarus* showed higher FPOM values (despite the adjustment for metabolic capacity) than the other two species when structure was present (Fig 3).

### Complexity measured as ‘amount’ and ‘fractal dimension’ in mono- and polycultures

We used data from 28 treatments to test for effects caused by species interactions (i.e. mono- *vs*. polycultures) and habitat complexity (measured as amount of structure and fractal dimension). Habitat complexity only significantly affected pH (Table 2, Collection B) and this effect was fully explained by the amount of structure added (2 or 3 plastic rings). The fractal dimension of the habitat was not a significant predictor, because the model ‘Fractal dimension’ did not explain more than the model ‘Amount’ in this collection of statistical models (Table 2). Average pH was lower in microcosms that had received two plastic plant rings compared to those with three plastic plant rings (data not shown), which might indicate a higher respiration rate at these habitat complexity levels.

Because we adjusted the number of individuals added to a microcosm according to their metabolic activity, species performed very similarly in the monocultures and measuring any change in their performance in polyculture was therefore straightforward. Leaf decomposition was not significantly different in our treatments (Table 2) but we observed that FPOM production was different for the three species and their combinations of two and three (confirming our observation for the monocultures [where it was almost significant]). For example, *Asellus* and *Gammarus* together had higher FPOM production than other species combinations but these high FPOM values were simply the average of the *Asellus* and *Gammarus* monoculture values (at the same complexity level). This meant that fitting the ‘Type’ model showed that no ‘true’ assemblage identity effects did arise because species still performed in an additive fashion (‘Type model’ significant; Table 2). In other words, assemblage identity would have been a significant predictor if ‘Type’ had not been fitted because there were certain species combinations that ‘performed better’ than others.

## Discussion

### Complexity effects on mono- and polycultures

We hypothesised that processes associated with leaf decomposition would increase with increasing complexity because more complex environments generally enhance foraging and feeding [11]. Indeed, microcosms with structure showed significantly higher FPOM and lower pH and this could be due to increased activity (e.g. higher respiration lowering pH) of individuals when their environment is more complex. However, other explanations are possible, such as slowed-down metabolism in microcosms without structure because individuals were stressed (this would explain both FPOM and pH results) or biofilm biomass on the structures contributed to respiration (this would explain the pH results but not FPOM and no biofilm was visible). Clearly there are future avenues for research in this regard and tracking growth, reproductive success, assimilation and respiration over multiple generations of species (feasible for microscopic metazoans for instance) in laboratory experiments offers a way to explore how the complexity of the environment and other factors (e.g. temperature) shape the performances and interactions of species. Respiration kits (e.g. optodes [30]) could be an ideal tool when linking respiration and pH for example.

Another hypothesis for this experiment was that complexity would be more important for processes than species interactions or identity *per se*. Indeed, while complexity influenced performance, processes were still largely additive, meaning that mono- and polycultures did not perform in an unexpected manner. One rationale for using *Cyclops* in these experiments was that we expected *Cyclops* to interact with the larger crustaceans by feeding on their faeces and not the leaves but we did not find any evidence for this here. In our experiments, the identity of the species was not an important predictor for leaf decomposition or FPOM. We expected the latter for leaf decomposition because we adjusted for metabolic capacity of each species and our previous findings show that, once body mass is accounted for, leaf decomposing species perform very similar [14,16].

### Statistics

When addressing habitat complexity on ecosystem responses, it is important to disentangle the effects of different measures of habitat complexity [6] and here we show that ‘more simple’ measures of habitat complexity (e.g. ‘structure present’ *v*s. ‘structure absent’) can override more sophisticated measures (e.g. ‘fractal dimension’)–when they are taken into account. In other words, if we had not considered ‘Structure present *v*s. structure absent’ and ‘Amount’, the more complex measure -fractal dimension of the structures- would have been a significant predictor of our response variables. However, it was not in our case because it was the most complex model in the ANOVA and did not explain more than the smaller models ‘Structure’ and ‘Amount’.

In this context, it is important to consider one feature of ANOVA: because the models are related and can be ordered in a hierarchy, ANOVA compares the goodness of fit of the linked models and tests for whether the difference between a model and its related smaller ones can explain the data significantly better or not. For example, ‘Assemblage Identity’ is a model related to ‘Richness’ (it is essentially a more complicated way to measure richness) and it can therefore only be significant if it explains more than ‘Richness’ [26].

### Outlook

In our design, we found a way to not confound ‘structure added’ with ‘complexity manipulated’ by nesting the two predictors. This approach is important for habitat alterations such as those used in river restoration projects and offers a way of more rigorous testing of the metrics (e.g. large woody debris or macrophyte cover) put in place. For example, it would be possible to find out if the amount of large woody debris added to a stream is more important than creating a ‘complex’ and ‘diverse’ environment for the river community. ‘Successful’ river restoration should deliver an increase in target species and ecosystem functioning (see [31]) but studies introducing structure to streams (e.g. wood, plants) have delivered inconclusive results in this regard [32]. There is certainly a need to monitor ‘restoration success’ [33] and small-scale experiments could be one very effective monitoring tool. Indeed, very controlled tests could be carried out by using plastic plants in the field -with a design similar to the one described here- and this would offer a way of testing measures effectively and objectively (e.g. if habitat complexity increases biodiversity in streams and rivers).

Laboratory experiments can provide more mechanistic explanations on ‘how complexity works’. An incentive for our experiment was that we expected that processes associated with leaf decomposition would increase with increasing complexity of the habitat because individuals would be in their preferred three-dimensional environment. The strongest effects in our experiments were observed between microcosms with and without structure, and the amount of structure explained all responses as well as fractal dimension explained it. This suggests that the complexity levels built were probably too fine and that a range of larger differences would have given stronger results. However, we used the same structures in functional response experiments and these show very clear differences in feeding efficiency of two aquatic, invertebrate predators (Flores et al. unpublished results). Indeed, short term experiments investigating the effects of habitat complexity typically focus on predator-prey relationships (e.g. [8]) because predators are either hindered [9,34] or aided [35] in their search for prey when structure is present and ‘complex’.

We believe that our experimental design and statistical approach can be used for future studies because it can disentangle the effect of different attributes of structure. We did not test for differences in dimensionality but we would expect that the most pronounced differences occur when consumers search for their resources in a three dimensional versus a two dimensional environment [11]. Pawar and colleagues developed a predictive model for 3D *vs*. 2D consumer searches that is backed up with empirical data and they show that consumers tend to encounter resources more frequently in three dimensions [11]. Therefore, complexity can shape processes via both traits and interactions.

Our short-term experiment with three species certainly demonstrated that habitat complexity is an important factor to be considered for a wide range of ecosystem processes. Out of the three processes measured in our study, one (leaf decomposition) was not affected by any of our predictors and our previous work has shown that measuring more than one process is needed to understand the importance of biodiversity and other factors (such as abiotic factors) for ecosystem functioning [16]. We conclude that consumer effects within one guild might often be additive when it comes to comparing single species to multispecies consumer assemblages but that habitat complexity has the potential to shape process rates, similarly to temperature.

## Supporting Information

S1 File R codes. R codes used to analyze the experiment with a worked example.
(DOCX)

S1 Table Data. An overview of predictors and responses.
(DOCX)

S2 Table Collection B ANOVA additional analyses. pH was measured throughout the experiment and ‘amount’ significantly influenced this response. Two examples are given here in the original R output. Comparing this to Table 2 shows how the ANOVA table was build.
(DOCX)
