# Supplementary material for: Habitat Complexity in Aquatic Microcosms Affects Processes Driven by Detritivores
Source: PLoS One. 2016 Nov 1;11(11):e0165065. doi: 10.1371/journal.pone.0165065 (PMC5089768; doi:10.1371/journal.pone.0165065)

**S1 File. R codes and example.** R codes used to analyze the experiment with worked example. Replace ‘**Response**’ with the response of interest, e.g. leaf decomposition per day or FPOM per day.

###exclude data to perform COLLECTION A: use only monocultures for ANOVA that testes for structure vs. no structure in collection A###

data <- subset(data,data$ID != "MC")

data <- subset(data,data$ID != "AC")

data <- subset(data,data$ID != "AG")

data <- subset(data,data$ID != "GC")

data <- subset(data,data$ID != "AGC")

**### ANOVA Collection A###**

lm1 <- lm(**Response** ~ Block + (S/A/F)*M, data = data)

summary(lm1)

anova(lm1)

plot(lm1)

### exclude richness level 0 and complexity 0 and microbial control for COLLECTION B###

data <- subset(data,data$R !="0")

data <- subset(data,data$F !="0")

data <- subset(data,data$ID != "MC")

**### ANOVA Collection B###**

attach(data)

Amount <-factor(A)

Amount

Fract <-factor(F)

Fract

Rich <-factor(R)

Rich

Block <-factor(Block)

Block

### now do the ANOVA for a specific **Response** ###

overallmean <-mean(**Response**)

overallmean

outType <-aov(**Response** ~x1+x2)

fitted(outType)

fitted(outType) - overallmean

summary(outType)

outAll <-aov(**Response** ~Block + (Amount/Fract)*(Rich +x1 + x2 +ID))

summary(outAll)

**Worked example for collection B**

Note the way in which fitted values are obtained for the model Type in Collection B; the fitted values for the model Type are not just averages. For the response pH, the fitted values for the model Type are

A 7.897 (see R output below, number 1-12)

G 7.945 (see R output below, number 13-24)

C 8.012 (see R output below, number 25-36)

AG 7.921 (see R output below, number 37-48)

AC 7.954 (see R output below, number 49-60)

GC 7.978 (see R output below, number 61-72)

AGC 7.951 (see R output below, number 73-84)

Note that the fitted value for A is not just the monoculture average. However, the fitted value for AG is 7.921, which is the average of 7.897 and 7.945, which are the fitted values for the relevant monocultures, which is precisely what the Type model says should happen.

R output
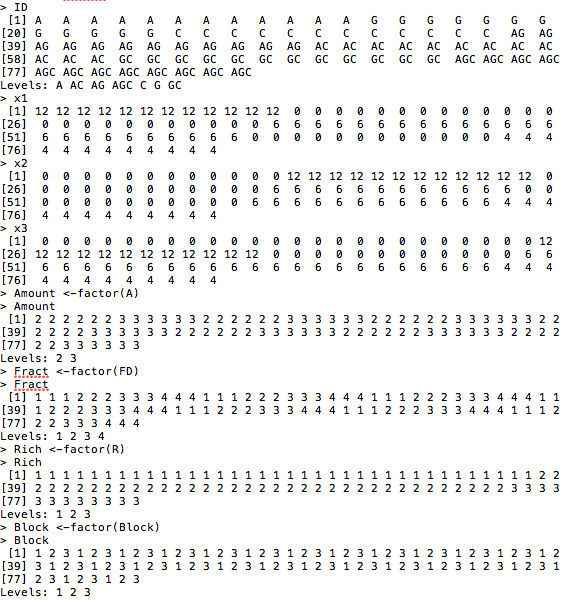


R output cont.


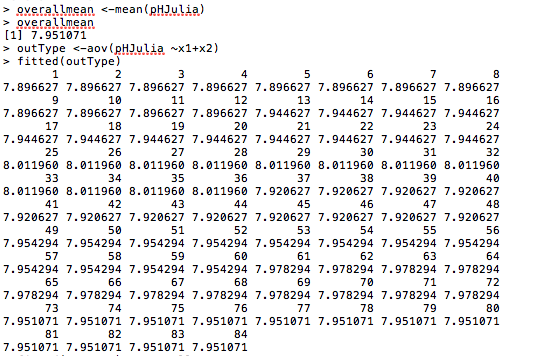


R output cont.


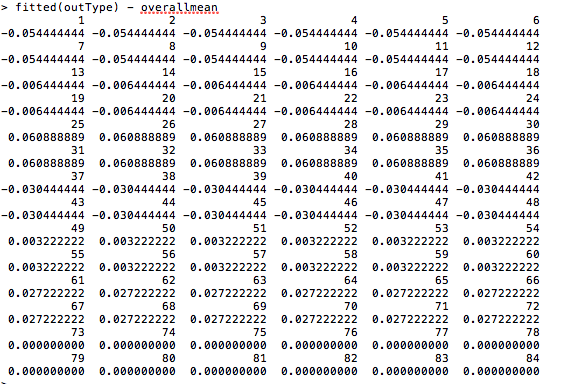


R output cont.


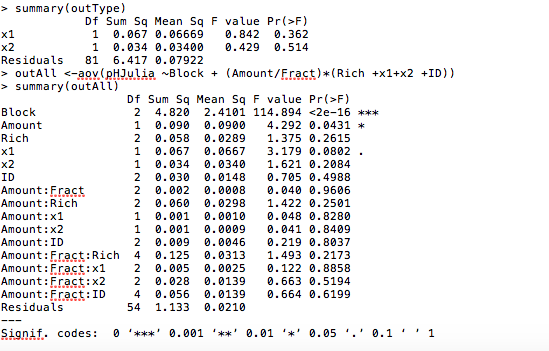

Supplement: S1 File — R codes used to analyze the experiment with a worked example. (DOCX) [file pone.0165065.s001.docx]
