# Supplementary figures and images for: Habitat Complexity in Aquatic Microcosms Affects Processes Driven by Detritivores
Source: PLoS One. 2016 Nov 1;11(11):e0165065. doi: 10.1371/journal.pone.0165065 (PMC5089768; doi:10.1371/journal.pone.0165065)

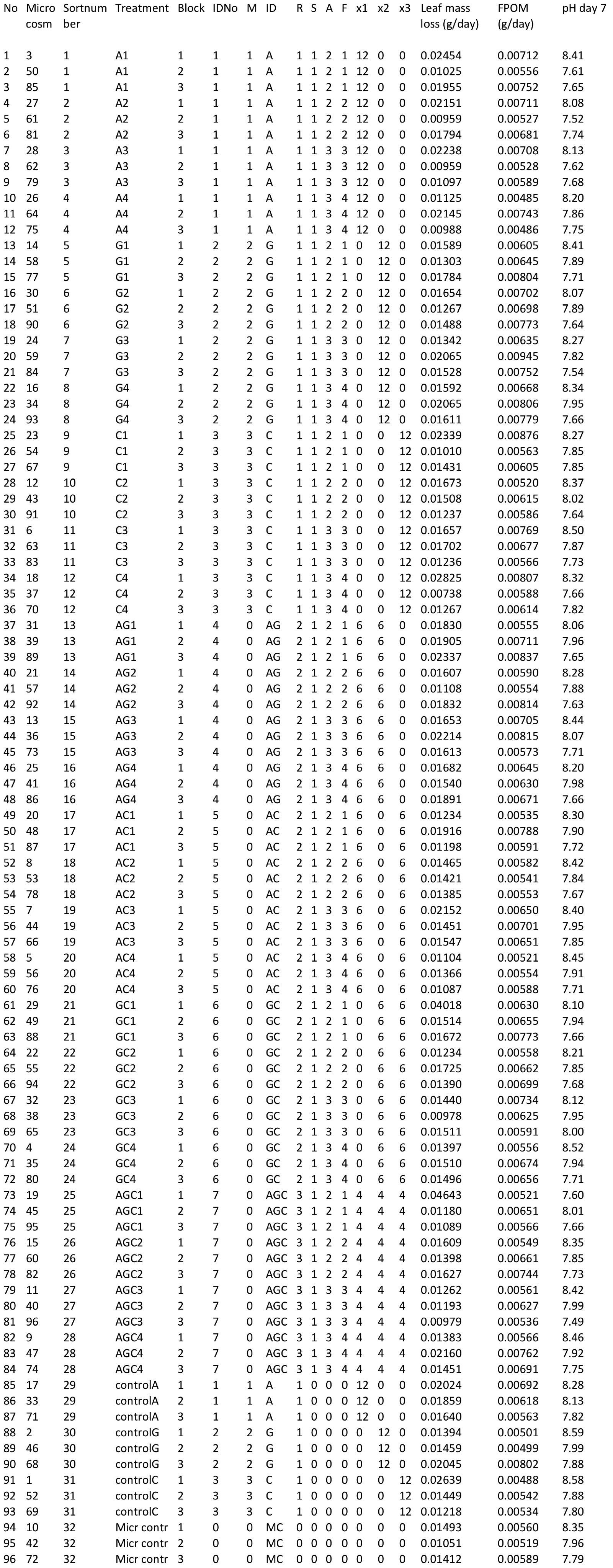
**S1 Table. Data.** This table gives an overview of predictors and responses.

Supplement: S1 Table — An overview of predictors and responses. (DOCX) [file pone.0165065.s002.docx]
