# Supplementary material for: Habitat Complexity in Aquatic Microcosms Affects Processes Driven by Detritivores
Source: PLoS One. 2016 Nov 1;11(11):e0165065. doi: 10.1371/journal.pone.0165065 (PMC5089768; doi:10.1371/journal.pone.0165065)

**S2 Table. Collection B ANOVA additional analyses.** pH was measured throughout the experiment and ‘amount’ significantly influenced this response. Two examples are given here in the original R output.


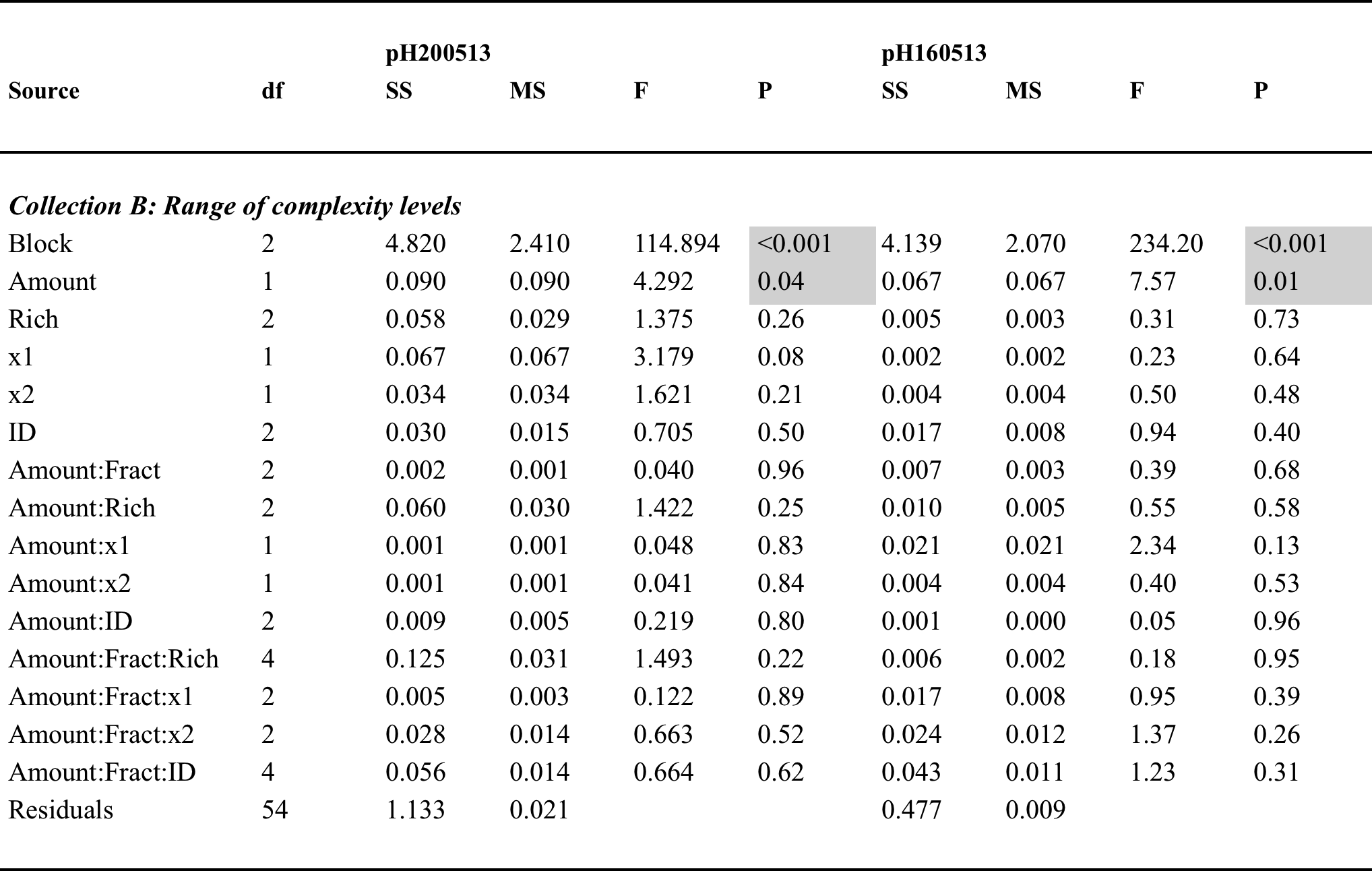

Supplement: S2 Table — pH was measured throughout the experiment and ‘amount’ significantly influenced this response. Two examples are given here in the original R output. Comparing this to Table 2 shows how the ANOVA table was build. (DOCX) [file pone.0165065.s003.docx]
